# The role of lipids in autophagy and its implication in neurodegeneration

**DOI:** 10.15698/cst2020.07.225

**Published:** 2020-05-19

**Authors:** Sergio Hernandez-Diaz, Sandra-Fausia Soukup

**Affiliations:** 1 Université de Bordeaux, Institut des Maladies Neurodégénératives, UMR 5293, 33000, Bordeaux, France.; 2 CNRS, Institut des Maladies Neurodégénératives, UMR 5293, 33000, Bordeaux, France.

**Keywords:** autophagy, neurodegeneration, lipids, sphingolipids, phosphoinositols, Parkinson's disease, Alzheimer's disease

## Abstract

Neurodegenerative diseases are, at present, major socio-economic burdens without effective treatments and their increasing prevalence means that these diseases will be a challenge for future generations. Neurodegenerative diseases may differ in etiology and pathology but are often caused by the accumulation of dysfunctional and aggregation-prone proteins. Autophagy, a conserved cellular mechanism, deals with cellular stress and waste product build-up and has been shown to reduce the accumulation of dysfunctional proteins in animal models of neurodegenerative diseases. Historically, progress in understanding the precise function of lipids has traditionally been far behind other biological molecules (like proteins) but emerging works demonstrate the importance of lipids in the autophagy pathway and how the disturbance of lipid metabolism is connected to neurodegeneration. Here we review how altered autophagy and the disturbance of lipid metabolism, particularly of phosphoinositols and sphingolipids, feature in neurodegenerative diseases and address work from the field that suggests that these potentially offer an opportunity of therapeutic intervention.

## INTRODUCTION

The prevalence of neurodegenerative disease like Alzheimer's (AD) and Parkinson's (PD) diseases will double in the coming years [1–3]. This is especially devastating since there is no cure nor effective treatment to stop the progression of most of these diseases. To revert this “no treatment/no cure” situation we need to gain a comprehensive understanding of the molecular mechanisms underlying the neurobiological basis of these diseases. Although neurodegenerative diseases are characterized by the appearance of very different symptoms caused by the particular disease's pathology, the abnormal accumulation of misfolded, dysfunctional and frequently aggregation-prone proteins is a commonly observed phenomenon [4–9]. On the other hand, lipids are also essential regulators of brain function and there is emerging evidence pointing out a role of lipids in neurodegeneration derived from ageing or neurodegenerative pathologies [10–15]. Although only the minority of neurodegenerative disease are caused by monogenic inheritance, studies using transgenic animals with the genetic variant of the disease have been very helpful in identifying common cellular and molecular mechanisms impacting the onset of neurodegenerative disease. After more than 20 years of research, failure of protein and lipid quality control mechanisms and neuronal homeostasis are placed in the centre of neurodegenerative diseases [16, 17]. In this context, autophagy has gained particular interest, since it can degrade cytosolic organelles and compartments [18–21] and has functional roles in lipid metabolism [22]. Moreover, lipids and lipid-binding proteins are essential components for the autophagosome formation and maturation. Emerging evidence shows that autophagy is altered in many neurodegenerative diseases and that defects in the autophagy pathway lead to the accumulation of dysfunctional and aggregation-prone proteins. Therefore, deciphering which step in the autophagy pathway is affected in a particular disease can shed light on the molecular mechanisms that lead to different pathologies and will provide an effective angle to develop disease-specific therapies that target defective steps in the autophagy pathway.

Lipids are essential components of biological membranes, but are also involved in cellular signalling processes such as autophagy [23–26]. Technological advances in the recent years have partially overcome the challenges present in investigating the role of lipids in biological processes [27–29]. In this review we want to highlight the function of lipids in the autophagy pathway with a focus on how alterations of lipid metabolism affect autophagy and the role of lipid metabolism in neurodegeneration. The function of lipids in neuronal homeostasis is starting to gather more attention but has not been discussed in great detail in connection with autophagy.

## AUTOPHAGY

**Figure 1:**
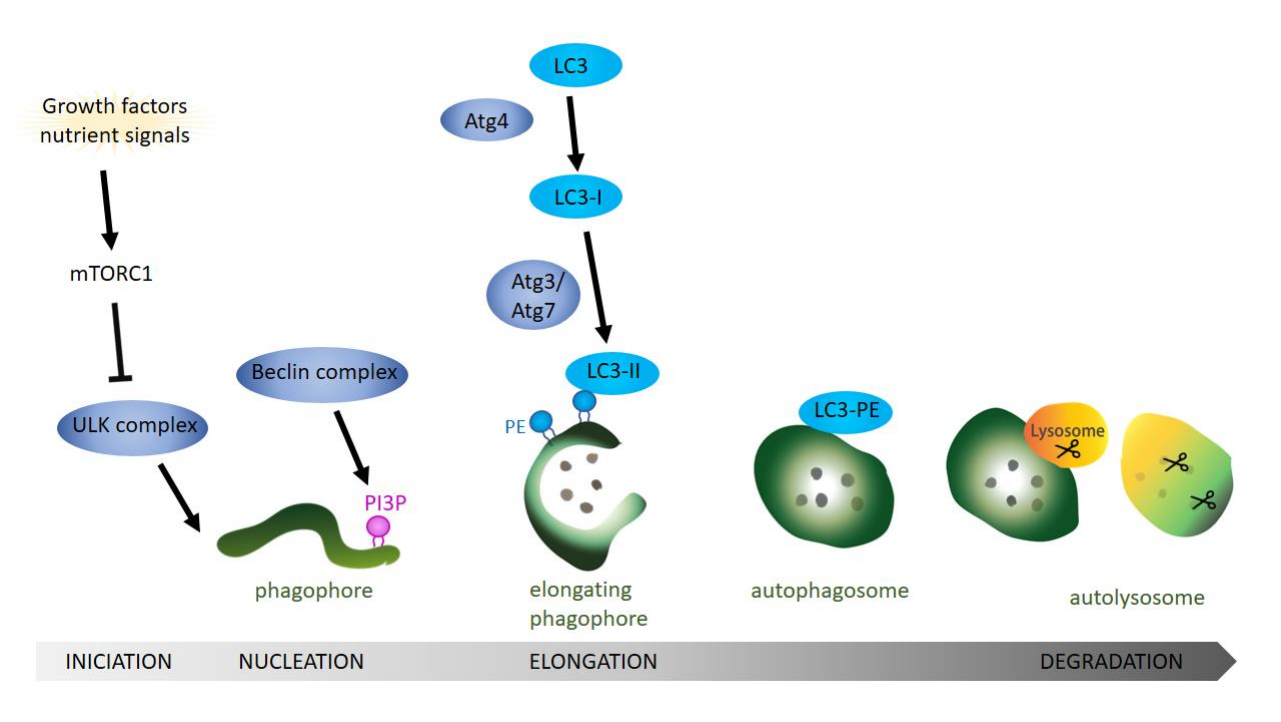
FIGURE 1: General overview of the autophagy pathway. Schematic drawing showing the autophagy process starting with the formation of the phagophore, followed by the completion of the autophagosome and finishing with the fusion of the autophagosome with the lysosome. Growth factors and nutrient signals inactivate the mammalian target of rapamycin complex 1 (mTOR1) leading to the activation and recruitment of the ULK complex to the phagophore. Activity of the Beclin complex leads to local enrichment of phosphatidylinositol-3-phosphate (PI3P). During the elongation of the phagophore, ATG4 processing of LC3 (ATG8 in Drosophila) and the subsequent conjugation to phosphatidylethanolamine (PE) on the phagophore membrane via ATG3 and ATG7 is essential step to form the autophagosome. After the fusion of the lysosome with the mature autophagosome, lysosomal proteases like cathepsin D degrade the autophagosomal content.

Autophagy is a catabolic pathway that degrades cytosolic content ranging from small particles to organelles like mitochondria. Three major autophagic pathways are known: micro- macro- and chaperon-mediated autophagy [30–34]. However, all three types converge within the lysosome to degrade cytosolic components and dysfunction in autophagy often leads to neurodegeneration [19, 20, 35]. In microautophagy the cytosolic components are translocated into the lysosome via direct invagination, protrusion or septation of the lysosomal membrane. In contrast, a selective degradation occurs during chaperon-mediated autophagy where proteins with a KFERQ-like motif are recognized and targeted for degradation by forming a complex with specific chaperones like HSC70 [36]. These complexes are then recognized by the lysosomal receptor LAMP2A and directly translocated across the lysosomal membrane towards the lysosomal lumen, where degradation takes place. The third type is macroautophagy, which is essential for the degradation of defective organelles, proteins, aggregates, and even microorganisms. This process starts with the sequestration of a portion of cytoplasm creating an initial structure named the isolation membrane or phagophore [37] (**Figure 1**). The expansion of the phagophore and its closure around the cytoplasmic material give rise to the autophagosome. To degrade the target material, autophagosomes fuse with endosomes, creating amphisomes which then fuse with lysosomes to generate autolysosomes [38–41]. Lysosomes provide the hydrolases required to digest the content and the internal membrane of the autophagosomes into basic metabolites that can be reused to obtain new cellular components or energy [42, 43]. In yeast, autophagic membranes originate at the Phagophore Assembly Site (PAS) but in higher eukaryotic cells the origin of the autophagic isolation membranes is still not completely clear. However, mitochondria, the endoplasmic reticulum (ER), endosomes, the Golgi body and even the cytoplasmic membrane have been proposed as sources of autophagic membranes [44]. The precise molecular mechanisms of the autophagy pathway has already been extensively reviewed [45–48]. Next, we will focus on the role of lipids in the regulation of different aspects of the autophagy pathway.

## THE ROLE OF LIPIDS IN AUTOPHAGY

**Figure 2:**
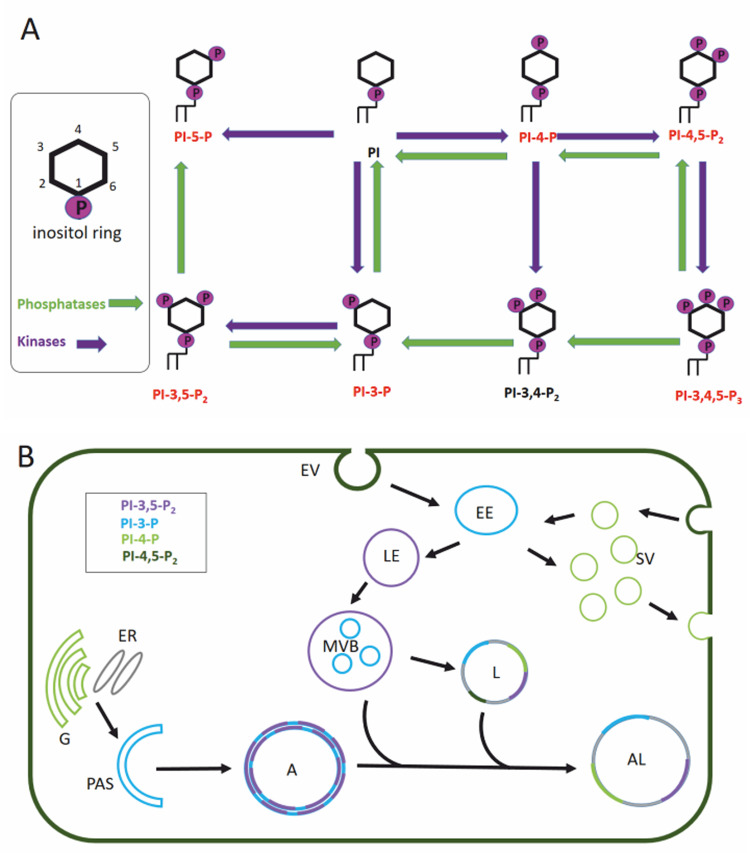
FIGURE 2: Phosphatidylinositol pathway and its role in cellular trafficking. **(A)** Phosphatidylinositols (PtdIns) are small lipids, consisting of two fatty acid chains, a glycerol backbone and an inositol ring. Kinases (violet) can phosphorylate the inositol ring at various positions (3, 4 and 5) leading to different phosphoinositides. We can differentiate between phosphatidylinositol monophosphates (PI3P, PI4P, and PI5P), diphosphates (PI3,4P_2_, PI3,5P_2_, PI4,5P_2_), and a triphosphate (PI3,4,5P_3_), that are substrates of different phosphatases (green) and kinases (see text for more details). Phosphoinositides that participate in the autophagy pathway are marked in red. **(B)** Membrane anchoring of the PtdIns is possible thanks to its glycerol backbone that positions the inositol ring towards the cytoplasmic site. Therefore, PtdIns often act as recognition motif for proteins containing a specific PI3P binding domain like pleckstrin homology (PH), FYVE, WD40 repeats, FERM, PTB, and PDZ to mediate signaling pathways including autophagy and clathrin-mediated endocytosis. Moreover, the specific enrichment of PtdIns can even mark the identity of intracellular organelles. Membranes are colored depending on postulated enrichment of specific phosphoinositols. PI4P enriched organelles are synaptic vesicles and Golgi, the phagophore and the early endosome are enriched in PI3P while the late endosome is enriched in PI3,5P_2_. The multivesicular body, a specialized late endosome, and the autophagosome are PI3,5P_2_ and PI3P positive organelles. Once the autophagosome fuses with the lysosome, the resulting autolysosome becomes enriched in PI3P, PI3,5P_2_ and PI4P. Golgi (G), endoplasmic reticulum (ER), phagophore assembly site (PAS), autophagosome (A), autolysosome (AL), multivesicular bodies (MVB), lysosome (L), endocytotic vesicle (EV), early endosome (EE), late endosome (LE), synaptic vesicle (SV).

Eukaryotic cell membranes are structured as lipid bilayers with glycerolipids, their phosphorylated derivatives, and sphingolipids as major components. Glycerophospholipids can be subdivided by their headgroups into five groups: phosphatidylcholine and phosphatidylethanolamine (PE) with a higher abundance and phosphatidic acid, phosphatidylserine and phosphatidylinositol (PI) with a lower abundance in the lipid bilayer. Phosphoinositides are phosphorylated phosphatidylinositols and have a major role in signalling events membrane trafficking (reviewed in [49, 50]). Depending on the phosphorylation of the inositol ring we can distinguish seven different phosphoinositides: PI, PI3P, PI3,5P_2_, PI5P, PI3,4,5P_3_, PI3,4P_2_, PI4,5P_2_ and PI4P (**Figure 2A**). The presence and composition of phosphoinositides in the membrane creates the membrane identity of the organelle (**Figure 2B**) and offers specific protein binding sites for signalling events [51, 52].

Membranes play a pivotal role in the autophagic pathway. Unfortunately, the precise lipid composition of the intermediates in the autophagy process (phagophore, autophagosome, amphisome, autolysosome) are still unknown. We are also lacking information on whether the different membranes from which autophagosomes derive generate autophagosomes with different lipid composition. This knowledge gap may be due to the fact that most studies focus on the role of proteins in the dynamics of the autophagic pathway. However, in addition to the well-studied lipid conjugation of light chain 3 (LC3)/ATG8, there is solid evidence that lipids such as phospholipids and sphingolipids also play a crucial role in the autophagy pathway.

### Function of glycerophospholipids in autophagy

#### The role of phospholipids in the initiation of autophagy

In mammals, the ATG1 orthologue ULK1 recruits and forms a complex with ATG13, FIP200 and ATG101 [53]. Next, the integral protein ATG9 is incorporated to the nascent autophagosome via its interaction with FIP200 [54]. The Beclin 1 complex, also known as phosphatidylinositol-3 kinase (PI3K) complex, that consists of Beclin 1, VPS34 (the class III phosphatidylinositol-3 kinase), VPS15 (mammalian orthologue p150) and ATG14 (ATG14L) [55] is then recruited to the ER to promote nucleation of the phagophore following ATG9 incorporation. The CXXC motif of ATG14L is crucial for localization of the ATG14L and thus the Beclin 1 complex in the ER [56] where ULK1 phosphorylation of Beclin-1 occurs, thereby enhancing the kinase activity of VPS34 [55].

Interestingly, the enrichment of phosphatidylinositol(3)phosphate (PI3P) by VPS34 in the membrane is a key event in autophagy nucleation [57, 58]. A locally enriched pool of PI3P serves to anchor proteins with PI3P binding motifs (see Review [59]), such as WIPI2 protein (WD-repeat protein interacting with PI), which is necessary to elongate and close the autophagic membrane [60–62]. PI5P is also involved in autophagosomal formation in PI3P depleted cells by binding WIPI2 during glucose deprivation [25]. This and other alternative autophagic pathways still require further research to fully understand the role of autophagy in health and disease.

#### The elongation of and closure of the phagophore relies on glycerophospholipids

Recent work discovered that the N-terminal region of ATG2 presents structural similarity to VPS13, a lipid transporter that functions at vacuole- ER and vacuole-mitochondria contact sites [63] and this conserved motif mediates the targeting of ATG2 to the ER and is indispensable for autophagy [64–66]. The authors showed that ATG2A can bind multiple glycerophospholipids and, in turn, transport them between membranes. While there is still controversy concerning the protein partners and the molecular mechanism that modulates the ATG2 lipid transfer, a recent paper established that GABARAP (γ-aminobutyric acid receptor-associated protein) is a crucial anchor protein for ATG2A whereas its binding to WIPI4 is not required in mammals for the formation and closing of the autophagosome [67]. Interestingly, other cellular compartments related with autophagosomes, like the endosomal compartment, are also enriched in PI3P and further research on the lipid transfer from these structures into the nascent autophagosomes would be necessary to fully understand the source of the autophagosomal membrane in different cellular compartments.

In the next step, the ATG16L1 complex, which includes the proteins ATG16L1, ATG5 and ATG12, is recruited to the pre-autophagosomal membranes. Structure-function analysis identified a conserved sequence within the coiled-coil domain of ATG16L1 that mediates its localization to pre-autophagosomal structures by direct binding with PI3P [68]. Moreover, in contrast to α-isoform, the β-isoform of ATG16L1 presents two membrane binding domains while the common N-terminal membrane-binding amphipathic helix is required for LC3B lipidation the β-isoform also has a C-terminal membrane-binding region that is dispensable for canonical autophagy but essential for VPS34-independent LC3B lipidation at perturbed endosomes [69]. The authors also demonstrate how the ATG16L1 C-terminus can sustain LC3 lipidation upon starvation also in the absence of WIPI2, indicating that these two isoforms may trigger autophagy in response to different cellular stimuli. The activity of the ATG16L1 complex promotes lipidation of LC3/ATG8 and GABARAP with PE [70–72]. Mutation in the lipid binding residues of ATG16L1 abolishes the conjugation/lipidation of LC3 to PE [68], which severely compromises the expansion and closure of the autophagosome, comparable with the phenotype observed in ATG8 mutants with the mutation G116A that abolishes any further lipidation. The ATG8 conjugation system is responsible for the lipidation of LC3/ATG8. This system works in a similar fashion to the ubiquitination system and comprises of the previously mentioned ATG5 and ATG12 together with ATG3, ATG4, and ATG7 proteins. ATG8 is cleaved by the cysteine protease ATG4 before being activated by ATG7 which acts like an E1 enzyme. ATG8 is then transferred to the E2 equivalent ATG3 prior to the conjugation of the PE that is carried by the ATG5-ATG12 complex acting as an E3 ligase [70, 73]. Interestingly, the precise moment in which the conjugation of ATG8 occurs could be controlled by the curvature of the autophagic membrane structure. *In vitro* studies showed that the highly curved rim of the growing phagophore attracts autophagic complexes and serves as a platform where most of the ATG8/LC3 lipidation takes place. These highly curved membrane sites are recognized by curvature sensing domains in ATG1, ATG3 and ATG14/Barkor [74–76]. Although lipid packing depends on the particular headgroup type, when the lipids are part of highly-curved biological membranes, they are mostly organized in a loose packing fashion on the outer (convex) surface. Interestingly, poorly hydrophobic motifs that are common in “curvature-sensing” domains require loose lipid packing in the bilayer to favour the insertion of protein motifs into the bilayer [77]. The group of Thomas J. Melia showed that the N-terminal domain of ATG3 contains an amphipathic helix that inserts into highly curved membranes by sensing loose lipid packaging. Moreover using a liposome reconstitution assay they could directly show that this mechanism was crucial for LC3 lipidation [75]. The precise molecular mechanism dictating how pre-autophagosomal membranes become highly curved is less well understood. Endophilin-A, a protein enriched at the axon terminals, has for long been known to deform membranes during clathrin mediated endocytosis to assist in the formation of newly endocytosed synaptic vesicles [78–81]. However, recently, Endophilin-A has also been implicated in autophagy at presynaptic terminals and this function is independent from its function in synaptic vesicle endocytosis [82]. LRRK2, a kinase that has been implicated in familial PD, phosphorylates Endophilin-A at Serine 75 within the central amphipathic helix of the N-BAR (Bin1/amphiphysin/Rvs167) domain [83]. This phosphorylation event changes the ability of Endophilin-A to interact with lipid bilayers [84]. The central amphipathic helix of Endophilin-A inserts deep into the acyl chains of the lipid bilayer, leading to a slight membrane curvature and the formation of tubules in an *in vitro* assay using giant unilamellar vesicles (GUVs). In contrast, phosphorylated Endophilin-A introduces a negative charge at Serine 75, leading to a less deep insertion of the amphipathic helix into the lipid bilayer. As a result the lipid head groups are pushed apart, similar to the above discussed lipid packing defects, creating highly curved membranes [84]. *In vitro* membrane reconstitution assays on GUVs demonstrated that phosphorylated Endophilin-A leads to the creation of small highly curved membrane bends that attract ATG3 [82]. Moreover, *in vivo* studies in *Drosophila* using transgenic animals showed that ATG3 recruitment to pre-autophagosomal membranes is also dependent on Endophilin-A phosphorylation state at the presynaptic terminal and that this is critical of ATG8 lipidation.

Production of PI3P is a critical step in autophagy. Thus, PI3P acts as a master regulator of autophagy via its enrichment in endosomal and autophagic structures. PI3P can be generated from different substrates. PI3Ks can phosphorylate PI to produce different phosphatidylinositol phosphates. Class III PIK3s, such as VPS34, a member of the Beclin 1 complex, can phosphorylate PI to produce PI3P [58] but this class of kinases can also use PI together with PI4P and PI4,5P_2_ to produce PI3P, PI3,4P_2_ or PI3,4,5P_3_ respectively. Therefore, modulation of these enzymes is expected to impact autophagosome formation. For instance, PI3K inhibitors, like 3-MA (3-methyladenine), can block autophagy by inhibiting autophagosome formation. However, how these enzymes and their enzymatic products and substrates are regulated during autophagy is not well understood. Interestingly, PI3K enzymes respond to insulin and other growth factors that also modulate autophagy and the PI3K pathway plays a crucial role in cellular and tissue homeostasis and disease [85]. On the other side, inhibition of phosphatases that dephosphorylate PI3,4,5P_3_ (such as PTEN) or the addition of synthetic PI3,4,5P_3_ can inhibit autophagy in cell culture [86]. Notably, the inhibition of autophagy caused by the loss of PTEN function does not affect the lipidation levels of ATG8/LC3.

Local production of PI3P due to class III PI3K activity has been proposed as one of the first steps necessary for the phagophore formation in mammals and other organisms [56]. This local concentration of PI3P may act as docking station for proteins with FYVE domains like DFCP1, GAPR1, WIPI or the *Drosophila* protein Zonda, that bind to PI3P [87]. These proteins are essential in the early steps of autophagosome formation and local concentration of PI3P is therefore critical for the initial steps of autophagosome formation.

PI3P function is also essential for the maturation of the autophagosome to generate amphisomes (autophagosome fused to endosomes) which, in turn, fuse with lysosomes to generate the autophagolysosome [88, 89].

Besides formation and maturation of autophagosomes and autophagolysosomes, PI3P lipids do also contribute to the autophagosome localization and transport. The mammalian motor protein FYCO1 binds PI3P through its FYVE domain and mediates plus-end microtubule transport of autophagosomes towards the endolysosomal system. Depletion of FYCO1 results in the perinuclear accumulation of autophagosomes [90]. Interestingly, this protein also interacts with LC3 and Rab7 and mediates anterograde transport of endocytic components via the kinesin Kif5 [91]. However, how the local concentrations of PI3P and FYCO1 precisely work in the connection of the autophagy and endosomal systems would require further investigation.

The role of PI3P is not restricted to bulk macroautophagy, as for instance PI3P is also necessary to localize Alfy, the mammalian orthologue of the *Drosophila* Blue Cheese, and TECPR1 in selective autophagy to eliminate aggregates and bacteria [92]. Interestingly, another phosphoinositide that may impact initiation of the autophagosome is PI4P, a lipid that recruits ATG13, a subunit of the ULK1 complex, to the nascent autophagosomes. Under starvation conditions, ATG9 positive vesicles enriched in BAR-domain containing proteins like arfaptins (ADP ribosylation factor interacting protein), and phosphoinositide metabolizing enzymes including the PI4K3 beta kinase facilitate the local production of PI4P [93].

#### Turnover of phospholipids is required for autophagosomal maturation

While the initial accumulation of PI3P on the phagophore is essential for the recruitment of some ATG proteins, the maturation step requires the removal of these ATG proteins from the autophagosome. Interestingly, a switch in the phospholipid composition in the nascent autophagosome is crucial for this protein removal. The first indication of this phenomenon came from yeast, where Ymr1 PtdIns3P phosphatase activity is required to turn over PI3P and to release the ATG proteins from the mature autophagosome [94]. The myotubularin phosphatase family (MTM) has emerged as a key family of phosphatases controlling PI3P levels and therefore controlling autophagy. For instance, the absence of MTM1 results in activation of mTORC1 [95] and several myotubularin-related phosphatases are reported to control different stages of autophagy, such as MTMR-14 / Jumpy controlling initiation steps in autophagosome biogenesis [96] and MTMR-3 regulating autophagosome biogenesis and size [97]. Unexpectedly, the ability of MTMR to control autophagy also relies on members whose catalytic domain is not functional. For example, the catalytically inactive MTMR-9 determines the enzymatic activity and specificity of MTMR-8 in autophagy in HeLa cells [98]. Further research addressing how these phosphatases regulate autophagy in neuronal cells and during neurodegeneration will be essential to better understand the importance of phospholipids turnover, autophagy and brain (dys)function.

**Figure 3:**
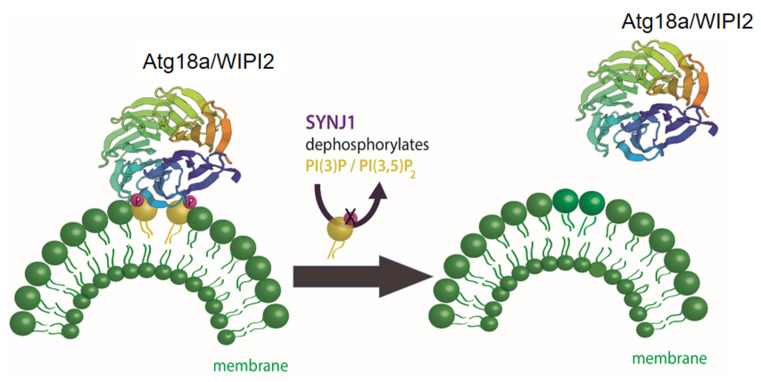
FIGURE 3: Maturation of autophagosomes relies on the turnover of PI3P/PI3,5P_2_ on autophagosomal membranes. In the initial phase of the autophagy pathway the accumulation of PI3P on the phagophore is required to recruit ATG18 and its mammalian homologue WIPI2. The WD40 domain in ATG18/WIPI2 folds into a seven bladed beta-propeller that contains two phenylalanine-arginine-arginine-glycine (FRRG) motifs in the sixth blade. This FRRG residue is a two sided recognition motif with binding specificity to PI3P/PI3,5P_2_. Maturation of nascent autophagosomes requires the shedding of autophagic factors like ATG18/WIPI2. At the presynaptic terminal, the phosphatase synaptojanin1 (Synj1) dephosphorylates PI3P/PI3,5P_2_ on autophagosomal membranes leading to the removal ATG18/WIPI2. Shedding of autophagic factors like ATG18/WIPI2 from the autophagosome is important for progression of the autophagic pathway.

The presynaptically enriched protein synaptojanin 1 (Synj1) is a lipid phosphatase well known for its function in synaptic vesicle endocytosis [29, 99–101] that has recently been shown to function in autophagy at presynaptic terminals [102]. Synj1 targets different polyphosphoinositides with its two distinct lipid phosphatase domains: 5-phosphatase which targets PI4,5P_2_ [103, 104] an important phosphoinositol in clathrin mediated endocytosis; and SAC1 which targets PI3P, PI4P, and PI3,5P_2_ [105], phosphoinositols with reported functions in autophagy. A PD causing mutation (R258Q) within the SAC1 domain of Synj1 [106–108] leads to the accumulation of immature autophagosomes at the presynaptic terminal in human neurons differentiated from patient-derived induced pluripotent stem cells (iPSC) [102]. Using transgenic *Drosophila* models of PD with a knock-in mutated Sac1 domain established that Synj1 removes the PI3P/PI3,5P_2_ binding protein WIPI2/ATG18a from immature autophagosomes (**Figure 3**). In synaptic vesicle endocytosis Synj1 hydrolyses PI4,5P_2_ causing the removal of adaptor proteins, including clathrin, from newly endocytosed vesicles. In autophagy, like in endocytosis, the release of the adaptor proteins from the organelle seems to be important for the maturation and trafficking of the organelle.

In brief, the presence and local production of the phospholipids like PI3P is critical to define the origin of autophagic membranes, as well as to recruit key protein complexes acting in autophagosome biogenesis, maturation and trafficking. The removal of specific phospholipids and/or their conversion to a different phospholipid species are also critical steps for the proper maturation and function of the autophagosomes.

#### The role of phospholipids in auto-lysosomal fusion

The presence of PI3,5P_2_ is low in late endosomes and lysosomes. In neurons, conversion of PI3,5P_2_ to PI3P by inositol polyphosphate-5-phosphatase-E (INPP5E) promotes lysosomal fusion with the autophagosome [89]. However, PI3,5P_2_ seems to have a very important role in these organelles regulating calcium signalling and autophagy through the TRPML1 ion channel [109]. While PI3,5P_2_ may work as an activator, another PI, PI4,5P_2_, seems to inhibit TRPML channels [110]. In this context it is worth mentioning that TRPML1 acts as a ROS (reactive oxygen species) sensor on lysosomal membranes involved in autophagy induction and that TRPML1 function is required for autophagosomal and lysosomal biogenesis [111]. Interestingly, cells from Niemann-Pick C (a lysosomal storage disease) disease's patients show reduced levels of activity in the TRPML1 and mutation of TRPML1 leads to the lysosomal storage disease, mucolipidosis type IV [112]. Interestingly, some lysosomal storage disorders are characterized by abnormal storage of various phospholipids due to reduced sphingolipid catabolism, but whether the local unbalance of phospholipid is a primary cause and how it may result in these disorders is still unclear.

The autophagosome–lysosome fusion event also relies on the GABARAP dependent recruitment of phosphatidylinositol 4-kinase IIα for PI4P production on autophagosomes [113]. On the other side PI4P conversion to PI4,5P_2_ on late endosomes leads to dissociation of Rab7 and, consequently, the release of pleckstrin homology domain-containing family M member 1 (PLEKHM1), a regulator of autophagosome-lysosome fusion [114].

**Figure 4:**
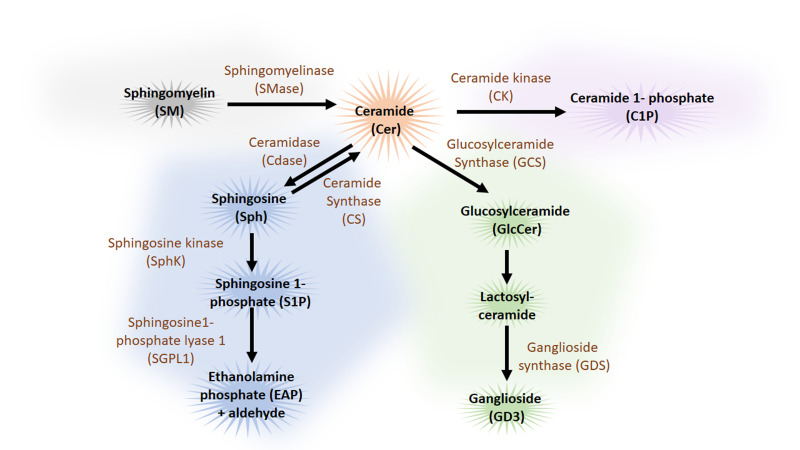
FIGURE 4: Overview of sphingolipid metabolism pathway. Ceramide (Cer) occupies a central position in the catabolic (blue), the sphingomyelin (grey) and hydrolytic (green) sphingolipid pathways. Apart from the *de novo* synthesis, ceramide can also be synthesized from sphingomyelin (SM) by sphingomyelinase (SMase) or from sphingosine (Sph) by ceramide synthase (CS). Cer can be catabolized to the biologically active metabolites, sphingosine (Sph) and sphingosine 1-phosphate (S1P) and further catabolized to ethanolamine phosphate (EAP) and C16 fatty aldehydes by ceramidase (Cdase) by the sphingosine kinase (SphK) and sphingosine1-phosphate lyase 1 (SGPL1) respectively. Cer can also be hydrolysed by glucosylceramide synthase (GCS) to glucosylceramide (GlcCer) and by ganglioside synthase (GDS) to ganglioside (GD3), a complex glycosphingolipid. Phosphorylation of Cer by the ceramide kinase (CK) produces ceramide-1-phosphate (C1P). Sphingolipids are coloured in black and enzymes are coloured in brown. (Note only autophagy relevant parts of the sphingolipid pathways are illustrated.)

### Function of sphingolipids in autophagy

**Figure 5:**
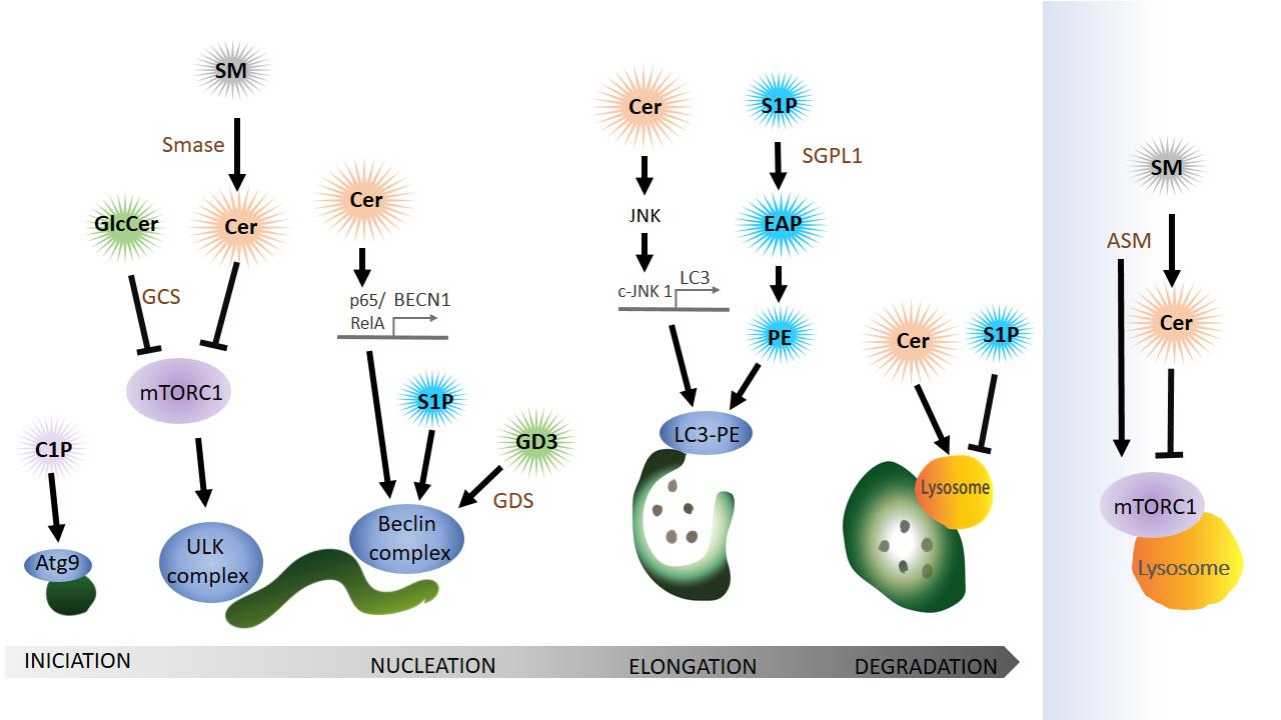
FIGURE 5: Bioactive sphingolipids, ceramide and sphingosine-1-phosphate (S1P) regulate autophagy especially in the initial steps. Lipids from the catabolic (blue), the sphingomyelin (grey) and hydrolytic (green) sphingolipid pathways function in the autophagy pathway. Sphingomyelin (SM) conversion to ceramide (Cer) by sphingomyelinase (SMase) affects mTORC1 activity on the lysosome and also on the phagophore. Cer can also induce autophagy by activating the transcription of ATG, BECN1 (Beclin) and LC3. Glycosphingolipids, glucosylceramide (GlcCer) and ganglioside (GD3) affect the autophagy pathway via mTORC1 or the Beclin complex respectively. LC3 conjugation to phosphatidylethanolamine (PE) is modulated by the catabolic sphingolipid pathway since sphingosine-1-phosphate (S1P) conversion to ethanolamine phosphate (EAP) that can be further converted to PE. During auto-lysosomal fusion Cer and SP1 have opposing effects (See text for details). Ceramide (Cer), sphingomyelin (SM), sphingomyelinase (SMase), acid sphingomyelinase (ASM), glucosylceramide (GlcCer), glucosylceramide synthase (GCS), ganglioside (GD3), sphingosine-1-phosphate (S1P), sphingosine-1-phosphate lyase 1 (SGPL1), ethanolamine phosphate (EAP), phosphatidylethanolamine (PE).

Sphingolipids are a family of lipids that use a sphingoid base as structural backbone. They are structural components of biological membranes where they can modulate the rigidity of membranes and participate in signalling to downstream effectors (for review see [115]). The *de novo* synthesis of sphingolipids starts at the ER and ultimately generates ceramide (Cer), a central component of sphingolipid metabolism. While sphingomyelinase (SMase) catalyses the generation of ceramide from sphingomyelin (SM), the enzyme glucosylceramide synthase (GCS) catalyses the synthesis of glucosylceramide (GlcCer) from Cer, the initial step of glycosphingolipid biosynthesis. Cer can also be deacylated to form sphingosine (Sph), which in turn is phosphorylated by sphingosine kinase (SphK) to sphingosine-1-phosphate (S1P) (**Figure 4**). Cleavage of S1P by the sphingosine 1-phosphate lyase1 (SGPL1) to fatty aldehyde and ethanolamine phosphate (EAP) is an exit from the complex sphingolipid network. Here, we will concentrate mainly on the bioactive sphingolipids Cer and S1P that have been shown to regulate mainly the initial steps of the autophagy process (**Figure 5**).

#### Sphingolipids acting at the stage of autophagy induction

Cer can initiate autophagy by lowering the uptake of nutrients like amino acids into the cell [116] leading to the activation of AMPK, upregulation of Beclin 1 and blocking mTORC1 [117]. Furthermore, Cer can also have an indirect role in autophagy initiation via GlcCer. Both pharmacological inhibition and siRNA-mediated reduction of the abundance of GCS, the enzyme that converts Cer to GlcCer, independently decrease AKT phosphorylation, thus blocking mTOR (mammalian target of rapamycin) and increasing autophagy levels [118].

Interestingly, a type of sphingomyelinase that respond to stress and amino acid deprivation is the acid sphingomyelinase (ASM), a class of SMase located at the lysosome that generates Cer from SM, leading to increased Cer levels. Furthermore, amino acid deprivation induces autophagy with an increase in ASM and Cer in human leukaemia cells, where knockdown of this enzyme suppresses autophagy induction [119]. However, increased ASM activity is also linked to abnormal autophagic degradation and brain pathology [120]. How ASM functions in the regulation of autophagy in neuro-degenerative diseases is still far to be completely understood. A recent publication shows that ASM is a negative regulator of autophagy via the lysosomal nutrient-sensing complex (LYNUS), a central multiprotein complex located in the lysosomal membrane that includes mTOR and the transcription factor EB (TFEB). LYNUS ensures cellular survival during amino acid deprivation and stress by regulating autophagy and protein synthesis. ASM control of the integrity of the LYNUS complex is required to maintain physiological functions of lysosomes. Notably, the reduction of ASM activity leads to the disruption of the LYNUS complex and activation of TFEB that then initiates the expression of lysosomal hydrolases and genes involved in autophagy while simultaneously reducing levels of Sph and S1P [121]. Taken together, although Cer is a positive regulator of autophagy, ASM abundance and activity can also impair autophagic degradation and physiological levels of this enzyme at the lysosome may prevent autophagy induction by controlling mTOR function at the level of the LYNUS complex.

#### Sphingolipids interactions during nucleation of autophagy

The ER-mitochondria contact site can function as a membrane donor for the phagophore. Lipid microdomains, so called lipid rafts, in ER-mitochondrial contact sites have recently been shown to be critical for this process. Ganglioside GD3, a building block of lipid rafts, associates with AMBRA1 and WIPI1, part of the Beclin complex, during autophagy initiation [122, 123]. The authors claim that GD3 in lipid rafts not only recruits autophagic factors but also contributes to membrane fluidity controlling this critical factor in the formation of highly curved membranes on the phagophore.

Cer can also initiate autophagy directly by activating the expression of Beclin 1 (BECN1) [117]. Copetti and co-workers stated that BECN1 expression is regulated by the NF-κB family member p65/RelA and consequently activation of p65/RelA enhances autophagy [124]. In line with these results another group showed that Cer can trigger p65 activation [125, 126]. In conclusion, Cer mediates the binding of p65/RelA with the κB binding site in the first intron of BECN1 and thereby activates the expression of BECN1. In addition to the Cer function in BECN1 expression, Cer also promotes dissociation of the Bcl2–Beclin 1 complex by the c-Jun N-terminal protein kinase 1 (c-JNK 1) [127]. Activation of c-JNK 1 leads to phosphorylation of a non-structural N-terminal loop of Bcl-2 that is necessary for the association with Beclin 1. Dissociation of Bcl-2 from Beclin triggers the induction of autophagy. At this point, it is important to highlight that ceramides can also function as soluble (or partially soluble) molecules when they are in the form of short-chain Cer. Indeed, because of their solubility in aqueous media, most *in vitro* experiments have used the short-chain forms as an exogenous source of Cer to investigate Cer's biological roles. For instance, the conversion of short-chain Cer into long-chain Cer by ceramide synthase is required to trigger autophagy [117] while it has been reported that treatment with exogenous short-chain ceramides and pharmacological treatments result in a comparable increase of endogenous Cer levels [116].

There is emerging evidence indicating that Cer activates gene expression of ATG genes like LC3. Experiments in the digestive tract of *Drosophila* showed that activation of the JNK pathway triggers autophagosome formation and a genetic interaction between JNK and ATG genes [128]. Moreover, inhibition of JNK signalling blocked Cer-induced autophagy and LC3 expression [129].

SP1 also positively regulates the Beclin complex but the precise mechanisms remain elusive. Overexpression of Sphk in primary neurons, leads to the production of SP1 and enhances the formation of preautophagosomal Beclin 1-positive structures and thus increases the formation of autophagosomes [130].

#### Elongation of the phagophore depends on sphingolipid interaction

In light of the debate about the origin of the donor membrane of the autophagosome, ATG9-positive vesicles may donate material to the elongating phagophore [131]. This stage can also be impacted by the availability of ceramide-1-phosphate (C1P), the phosphorylated form of Cer. C1P promotes liposome fusion [132] and might enhance the formation of Golgi-derived ATG9 vesicles that subsequently fuse at the phagophore [133]. Counteracting the activity of S1P, SM accumulation in recycling endosomes impairs ATG9 trafficking and as a consequence preautophagosomal structure cannot mature to form an autophagosome [134].

S1P levels can have a dramatic effect on autophagosomal formation by affecting the lipidation of LC3 to the autophagosomal membrane. Developmental depletion of sphingosine-1-phosphate lyase 1 (SGPL1) specifically in the nervous system leads to increasing levels of S1P without affecting other sphingolipid metabolites like Cer or SM [135]. S1P cleavage by SGPL1 into EAP and aldehyde is considered an exit point of the sphingolipid pathway. Phosphate cytidylyltransferase 2 (PCYT2) can than convert EAP to CDP-ethanolamine and subsequent reaction by a methyltransferase leads to the production of PE [135]. A post-translational lipidation reaction can then conjugate LC3 to PE on the phagophore. This article indicates that restoring physiological PE levels, either by adding exogenous PE or using pharmacological approaches, could be used to rescue autophagy-defects caused by PE deficit. Indeed, addition of PE can reduce the accumulation of aggregate prone proteins such as APP (amyloid precursor protein) and SNCA (α-synuclein) in primary cultured neurons and in hippocampal slices from conditional SGPL1 knockout (KO) mice, indicating that PE supplementation may be a suitable therapeutic approach [135].

#### The function of sphingolipids in the degradation of autophagosomal cargo

Even though sphingolipids have not been shown to directly promote fusion of the autophagosome with the lysosome to form the autolysosome, various studies have implicated sphingolipid metabolites in the degradative step of the autophagy pathway. Some sphingolipids like Cer with a chain length C18, can mediate target-specific degradation of mitochondria by autophagy [136, 137]. Cer on mitochondria can bind lipidated LC3 on autolysosomal membranes and promote mitophagy [138].

Sphingolipids can mediate the degradation of particular targets, but they also serve as autophagic substrates. This has been observed in epithelial cancer cells where K-Ras mediates autophagic S1P degradation [139]. Blocking autophagy in these cancer cells restores S1P localization and apical extrusion, an apoptotic process that maintains epithelial barriers and suppresses the spreading of cancer cells into the surrounding tissue.

In brief, sphingolipids like Cer or SP1 play an important role in autophagy by regulating autophagosome biogenesis, maturation and autophagosome-lysosome fusion, a critical step for the degradation of the autophagosome cargo.

## AUTOPHAGY AND NEURODEGENERATION: A LIPID PERSPECTIVE

The mutation of genes involved in the regulation of autophagy are frequently associated with neurodegenerative diseases (review in [140]). Studies using KO models of core autophagy genes (atg5 and atg7) in flies and mice showed that autophagy is critical for neuronal survival [19, 141, 142]. Conditional KO of atg5 in neurons causes motor dysfunction and abnormal accumulation of proteins and aggregates in the cytoplasm [141]. Loss of ATG7 specifically in the central nervous system of mice also impairs motor function and leads to neurodegeneration and early death. Interestingly, two studies showed that these mice accumulate polyubiquitinated aggregates, a hallmark of many neurodegenerative diseases caused by autophagy dysfunction. In addition, these mice also accumulate abnormal levels of SNCA and LRRK2 [20], the two major components of the PD-associated Lewy bodies [4, 5]. Moreover, variations in the locus of atg5 and atg7 are risk factors for PD [143, 144]. However, the precise molecular mechanism occurring between defective autophagy and the onset of neurodegeneration remains elusive.

As previously mentioned, the appearance of protein aggregates in the brain is a hallmark of many neurodegenerative diseases including AD, PD, amyotrophic lateral sclerosis, and Huntington's disease (HD). While the amount of protein aggregation correlates positively with neurotoxicity, the importance of understanding clearance mechanisms in neurons became evident when Yamamoto *et al.* demonstrated that a constant influx of the pathological huntingtin protein was required for the disease, indicating that clearance of these aggregates could alleviate symptoms of neurodegenerative diseases [145]. Later, Hara *et al.* demonstrated that autophagy was one of these essential clearance mechanisms to prevent accumulation of inclusion bodies in neurons [141]. Autophagic clearance of protein aggregates requires cargo receptors like sequestosome 1 (SQSTM1/p62), NBR1, optineurin (OPTN), and NIX/BNIP3L, that sequester these aggregates to the autophagosome [146–149]. Several aggregate prone proteins, like ataxin-1 (SCA1) and ataxin-3, polyglutamine (polyQ)-repeated huntingtin, aggregate-prone α-synuclein A53T and A30P mutants, tau and SOD1 (copper/zinc superoxide dismutase) are predominately degraded by the autophagy pathway [150–154].

Autophagy plays a crucial role in neural homeostasis since specific suppression of autophagy in the central nervous system leads to neurodegenerative phenotypes [19, 141] and disruption of this pathway in dopaminergic mice neurons promotes presynaptic accumulation of “Parkinson's proteins” like LRRK2 and α-synuclein [155].

Interestingly, macroautophagy is a central process in the clearance of neuro-pathological aggregates in several neurodegenerative diseases. For instance, macroautophagy reduces the toxic level of aggregates caused by the accumulation of mutant huntingtin, α-synuclein and tau *in vitro* as well as *in vivo Drosophila* and mouse disease models [150, 153, 156, 157].

### Function of phospholipids in neurodegeneration

We have described how phospholipids are important for the biogenesis of autophagosomal structures. Hence, regulation of local levels of the different phospholipids specimens is important for this early autophagosome biogenesis. For instance, autophagosome formation can therefore be positively or negatively regulated by PI3P kinase complex or MTMR phosphatases respectively.

#### Phosphoinisitol metabolism in amyotrophic lateral sclerosis and Charcot-Marie-Tooth disease

Mutations in the phosphoinositol(3,5) bisphosphate 5' phosphatase Fig4 lead to neuronal degeneration in rodent brains and has been associated to neurodegenerative disease in humans, including a recessive form of Charcot-Marie-Tooth disease type 4J and amyotrophic lateral sclerosis [158]. Likewise, mice with a mutant Vac14, one of the enzymes required for PI(3,5)P_2_ synthesis, also suffer from the loss of neurons [159]. These defects could be associated with the alterations of the autophagic pathway, since Fig4 and Vac14 mutant mice show accumulation of lipidated LC3 - a hallmark of autophagy activation - and LAMP2 in neurons and astrocytes [160]. Noticeably, the accumulation of p62 and ubiquitinated proteins is more prominent in the brain regions that show high neuronal death. However, how Fig4 regulates autophagy is still far from being understood. For instance, Fig4 does also regulate lysosome size independent of the phosphatase activity in muscle of invertebrate models [161]. However, this non-catalytic function of Fig4 has not been yet investigated in neurons or mammalian models of the disease.

#### Deregulation of phospholipids in HD

Decreasing the activity of PIP4Kγ in mice primary neurons expressing mutant huntingtin protein in human patient fibroblasts leads to an increase in PI5P, PI3,5P_2_ and PI3P levels that positively stimulate basal autophagy and the degradation of aggregates and polyQ proteins. This is particularly interesting since pharmacological inhibition of PIP4Kγ with the compound NCT-504 could potentially have therapeutic applications [162]. Moreover, the overexpression of huntingtin can stimulate autophagy and endo-lysosomal systems in neurons [163] and the polyQ expansion in the pathological form of huntingtin can change the phospholipid binding affinity of the huntingtin protein [164] which may explain why huntingtin localizes to endo-lysosomes and autophagosomal structures in neurons from HD patients [163]. Further research is necessary to understand if mutant huntingtin pathology can, at least partially, resided at the level of phospholipid interaction.

#### Dysfunction of phospholipids in PD

Lipidomics of human primary fibroblasts from PD patients with a Parkin mutation, revealed that gangliosides, PI and phosphatidylserine levels were increased [165]. The authors suggest that this could be the result of autophagic alterations that have been extensively studied in Parkin mutant animal models. Although these results were not obtained in brain samples, the ubiquitous function of Parkin within the mitochondria may indicate these levels are also increased in the brain. Contrary, another report indicates that mitochondria from aged Parkin null mice brain show reduced levels of diverse types of phospholipids [166]. However, the mechanisms acting at pathophysiological level for defective phospholipid homeostasis in PD patients harbouring Parkin mutations need to be further studied.

As mentioned before, mutations within the SAC1 domain (R258Q and R459P) of Synj1 inhibit the phosphatase function of the SAC1 domain and cause early onset PD [106–108]. Knock-in flies with the R228Q mutation in Synj1 (the corresponding pathogenic mutation to R258Q in flies) show age-dependent neurodegeneration of dopaminergic neurons, activity-dependent neurodegeneration of photoreceptors and accumulation of immature autophagosomes [102], indicating that the function of the SAC1 domain is necessary for autophagy and required for neuronal survival. The function of the synaptic protein Endophilin-A is also critical for neuronal survival in mice and flies [80, 82, 167] and variation at the Endophilin-A1 locus (SH3GL2) is proposed to be a risk factor for PD [168]. We have already discussed that the Parkinson protein LRRK2 phosphorylates Endophilin-A and that insertion of phosphorylated Endophilin-A into the lipid bilayer leads to more wedging of the lipid head group and creates highly curved membranes that attract ATG3. Interestingly, similar to Synj with the R228Q, phosphomimetic and phosphodead Endophilin-A also leads to neurodegeneration of dopaminergic neurons and photoreceptors [82].

#### The role of phospholipids in AD

Progressive loss of memory, the appearance of amyloid β (Aβ) aggregates in plaques, and hyperphosphorylated tau as neurofibrillary tangles in the brain are all hallmarks of AD, the most common neurodegenerative disease. Post mortem analysis of AD patient brains revealed a reduction of PIP_2_. The authors of this study claim that this reduction was a result of increased levels of Synj1 that is known to dephosphorylate PIP_2_ during clathrin-mediated endocytosis [169]. Another study showed that deregulation of PIP_2_ levels in AD mice models has an effect on neurotransmission, spatial learning, and memory [170]. Although the idea of using phospholipid levels as diagnostic or prognosis readout have been explored, there are evidences suggesting that changes of phospholipids are not detected in early stages of the disease [171] although screening using state-of-the-art lipidomics of blood or cerebrospinal fluid would be worthy to explore.

Genome-wide association studies have recently identified PICALM/CALM (phosphatidylinositol binding clathrin assembly protein) loci associated with increased AD development risk [172]. PICALM directly interacts with PIP_2_ on the plasma membrane during clathrin mediated endocytosis. Genetic evidence of PICALM as a risk factor for AD was further confirmed by revealing that the adaptor complex AP2 and PICALM interact with LC3 to mediate the degradation of Alzheimer's C-terminal fragment APP via autophagy [173]. Moreover, Zebrafish and *Drosophila* tau models showed that altered CALM levels enhance neurotoxicity by inhibiting autophagy. Functionally, CALM regulates lipid uptake from the plasma membrane to the phagophore and down regulation of CALM disrupts the degradation of tau by autophagy leading to accumulation of tau aggregates.

#### Friedreich's ataxia

Friedreich's ataxia is an autosomal recessive neurodegenerative disease caused by decreased expression of frataxin, a mitochondrial protein, leading to mitochondrial dysfunction [174, 175]. However, autopsy reports from patients showed reduced PE, phosphatidylserine and linoleic acid levels in the brain [176]. Moreover, the GAA triplet repeats in frataxin also affect the adjacent gene PIP5KB leading to decreased PI4,5P_2_ levels [177]. Although autophagy alterations have been connected to Friedreich's ataxia, the precise molecular mechanism remains enigmatic [178].

### Function of sphingolipids in neurodegeneration

Alterations in sphingolipid metabolism are associated with many neurodegenerative diseases. This is not solely linked to the role of sphingolipids as important components of membranes but also to the bioactivity of some sphingolipids, functioning as cell signalling molecules in a variety of biological processes. Familial forms of neurodegenerative lysosomal storage disorders (LSD) like Gaucher, Krabbe, Niemann-Pick type 1 and Fabry are caused by mutations in genes encoding enzymes that function in the sphingolipid pathway [179–181]. Lysosomes are essential components for the degradation of the autophagosome cargo. Actually, autophagy defects are commonly present in LSDs (review in [182]). This points out the difficulties to segregate pure autophagy defects (e.g. in autophagosome biogenesis or maturation), from lysosomal defects affecting autophagy (e.g. inhibiting the autophagic flux).

It is thought that increased levels of sphingolipids and their byproducts can be neurotoxic [183]. However, the precise molecular mechanisms underlying this neurotoxicity is not well understood. Furthermore, alterations of sphingolipid levels that are not caused by mutations in genes encoding for enzymes of the sphingolipid pathway have also been documented in Alzheimer's, Parkinson's, and multiple sclerosis.

#### Alteration in SP1 and Cer in neurodegenerative disease

As early as in the 1960's researchers had established a link between aging and sphingolipid metabolism. Levels of GlcCer increase in the brain with aging and strikingly AD brains show abnormally elevated levels of GlcCer [184]. Years later, research about sphingolipids in aging and AD brains focused more on Cer and S1P. A balance between Cer and S1P, a so-called “Cer/S1P rheostat”, defines the equilibrium between the pro- and anti-apoptotic forces in neurons [185–187]. Furthermore, increased levels of S1P in neurons have a neuroprotective function via the induction of autophagy by benzoxazine. This neuroprotective role was neuron specific, since benzoxazine did not induce autophagy in astrocytes [188].

#### The function of sphingolipids in degradation of aggregate-prone proteins

The accumulation of Aβ as aggregate deposits has been linked to decreased autophagic flux in Alzheimer's patients and increasing evidence places autophagic alterations in the centre of AD pathology. Various reports showed that ASM, which negatively regulates autophagy, is upregulated in brains of Alzheimer's patients [189, 190] and that Aβ levels correlate with ASM activity [190]. Inhibition of ASM in AD mice lead to reduced Aβ aggregates and improved memory performance. Furthermore, reducing ASM levels in human iPSC-derived neurons from Alzheimer's patients restores autophagic flux via defects in lysosome biogenesis [191].

Dysregulation of glycosphingolipid metabolism has also been reported in PD. Clinically, PD is diagnosed by the onset of motor symptoms including rigidity and tremor. Post mortem, PD is defined by the loss of dopaminergic neurons and the increased appearance of PD prone proteins including full length SNCA, and the C-terminal fragment of APP in the brain compared to wild type mice. As already mentioned, SGPL1 mutant mice show decreased levels of PE that functions as an anchor for LC3 on the phagophore and consequently impairs LC3 lipidation, leading to accumulation of phagophore-like structures and inhibiting autophagosomal maturation. Accordingly, pharmacological or genetic inhibition of SGPL1 blocks autophagy and induces aggregate accumulation while the addition of PE restores autophagic flux and controls levels of APP and SNCA in primary mouse neurons. Not surprisingly, neuron-specific depletion of SGPL1 in mice leads to impairment in memory and learning. These cognitive deficits were observed using a Morris water maze to analyse spatial learning and memory but also in an associative learning and memory test [135]. Moreover, mutations in glucocerebrosidase (GBA) results in increased GlcCer levels in lysosomes, thus affecting the degradation of SNCA by the lysosome [192]. Indeed, the risk of developing PD is increased five-fold even when only one GBA allele is mutated [193]. Work in primary neurons has further implicated sphingolipids in SNCA-associated pathology underlying PD. Mutant SNCA levels were reduced in primary neurons treated with a GlcCer synthase inhibitor that led to increased autophagy flux [118].

There is also an intriguing link between Cer accumulation and retromer disruption in Parkinson's fly models for PLA2G6, VPS35 and SNCA [194]. The authors found that PLA2G6 loss of function mutation affects Retromer function via the stability of its subunits; VPS35 and VPS29. This in turn affects retrieval of membrane bound sphingolipids via the endolysosomal system, leading to the accumulation of Cer that enhances further lysosomal dysfunction.

## CONCLUSION

**Figure 6:**
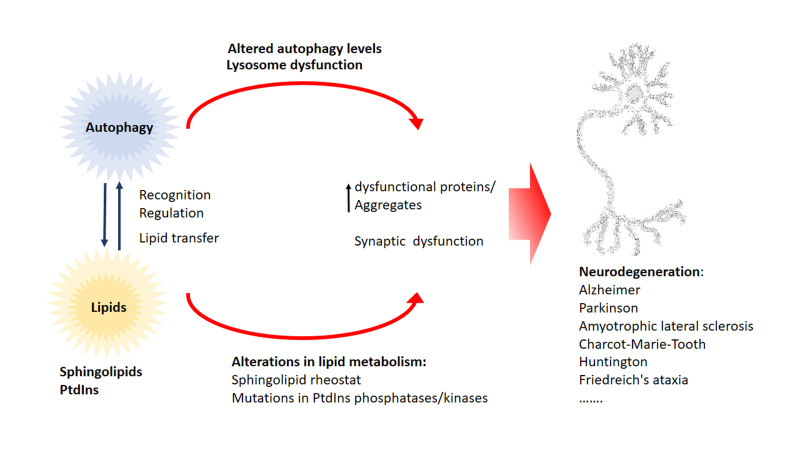
FIGURE 6: Proposed link of lipids in autophagy and neurodegeneration. There is a tight relationship between lipid metabolism and autophagy. Various sphingolipids function as bioactive lipids in the regulation of autophagy, while phosphoinositide phosphates (PtdIns) mainly function in the recruitment of autophagic factors. Autophagy is known to be an essential physiological mechanism to degrade dysfunctional and aggregate-prone proteins that are commonly accumulated in neurodegenerative disease. Changes in the sphingolipid rheostat (imbalance in the ratio between the concentrations of the apoptosis activator ceramide, and the anti-apoptotic mediator sphingosine-1-phosphate) have been implicated in many neurodegenerative diseases. Deregulation of phosphatidylinositol levels lead to neurodegeneration and various phosphatases and kinases targeting phosphatidylinositols have been found to be mutated in various neurodegenerative diseases.

In this review we highlighted the function of lipids in various stages of the autophagy pathway, where they can act as recognition motif to attract regulatory autophagy complexes, to regulate the autophagy core machinery as bioactive lipids or even by regulating transcription of ATGs (**Figure 6**). Moreover, alterations of the lipid metabolism leads to defects in the autophagy pathway, illustrating the tight connection between lipid supply and the formation/progression of the autophagosome.

Currently available evidence suggests that the accumulation of ubiquitin-positive protein aggregates is a common event in different neurodegenerative diseases like Alzheimer's, Parkinson's, amyotrophic lateral sclerosis, Charcot-Marie-Tooth, Huntington's and Friedrich's ataxia. Autophagy is one of the major pathways that is capable of degrading misfolded proteins and aggregates and therefore autophagic alterations lead to the accumulation of aggregates and there is ample evidence in literature showing that alterations of the autophagy pathway promote neurodegeneration. Not surprisingly, restoring autophagic defects in neurodegenerative disease has gathered special attention as a therapeutic target. On the other side, alterations in lipid metabolism have been connected to neurodegeneration and the accumulation of aggregates. In recent years, mutations in various phosphatases and kinases that target specific PtdIns have been discovered in amyotrophic lateral sclerosis, Charcot-Marie-Tooth disease, Huntington's and Parkinson's patients. Furthermore, not only alterations in PtdIns pathway but also in sphingolipid metabolism have been tightly connected to neurodegenerative diseases. For instance, changes in the sphingolipid rheostat are implicated in the most common neurodegenerative diseases Alzheimer's and Parkinson's. Sphingolipid rheostat is defined by the reciprocal role of the lipids Cer and S1P. In this model S1P has a pro-survival role while Cer is thought to play a pro-apoptotic role. The sphingolipid rheostat has not only been discussed in the context of neurodegeneration and physiological homeostatic processes but also in the regulation of autophagy, an essential process in many (patho)physiological events. Besides the importance of sphingolipid rheostat in pathology, homeostasis, and autophagy we are still not certain of the underlying mechanisms of this process. We propose a direct link between dysfunction of lipid metabolism and autophagic alterations that ultimately leads to neurodegeneration. Alterations in autophagy and lipid metabolism have been independently linked to neurodegenerative disease rather than as part of a unique functional network. Alterations of the lipid metabolism have a direct impact on the autophagic pathway and through this could impact neuronal and synaptic function, leading to the accumulation of dysfunctional proteins and aggregates that promote neurodegeneration. Currently, targeting of both the autophagy pathway and especially the sphingolipid pathway are seen as a therapeutic opportunity for various neurodegenerative diseases (reviewed in [195]). However, a much more comprehensive understanding of the interaction of lipids with autophagy is required to elaborate predictive models that would permit evaluation of side effects in neuronal homeostasis over potential benefits. The fact that the lipid composition of the brain changes during ageing, a major risk factor for neurodegenerative diseases, is not new (for review [196]). However, a comprehensive characterization of this lipid changes in a cell-type fashion during the neurodegenerative process is still incomplete. Moreover, we are still missing the full picture of the dynamics of the autophagosomal structures and their lipid composition at a cellular and subcellular (e.g. cell compartments) levels. Emerging techniques like correlative light and electronic microscopy (CLEM) may help to better understand the ultrastructure of autophagosomal structures in a cell and even compartment specific manner. However, the precise identification of the lipid composition of autophagosomal membranes is still a challenge.

Yeast is a valuable organism for autophagy research, however, there are likely differences in the autophagosome lipid composition between yeast and mammals [197]. These differences may be further complicated by the potential differences between the autophagosomes of mammals originating from different organelles. Moreover, the therapeutic regulation of autophagy via the manipulation of the local lipid composition first requires a better understanding of the mechanisms by which autophagy can dictate cell death or survival in neuronal cells. This balance between cell death and survival is a critical issue in neurodegeneration and also in cancer. For instance, the idea of targeting sphingolipid metabolism (such as Cer) as a therapeutic approach has been investigated in cancer research, but progress is limited partially due to an incomplete knowledge of the interplay between Cer and autophagy. There are a few drugs in different clinical trial phases aiming to target the lipid metabolism (for a review see [198, 199]), the development of drugs to specifically modulate autophagy by targeting the PI and especially the sphingolipid metabolisms is yet a nascent field. An improved understanding of how lipids regulate basal and induced autophagy in healthy individuals and during neurodegenerative conditions will be essential to identify better therapeutic molecules targeting lipid metabolism and autophagy. A pharmacological manipulation of lipid metabolism in neurons and glia may not only be useful for neurodegenerative disorders but for a larger set of neurological conditions. For instance, a possible role for autophagy [200] and lipid alterations [201] has been identified in animal models of DYT1 primary dystonia.

Advances in the field are right now limited by the current means to intervene in lipid pathways and in the detection of specific lipid species *in vivo*. Technological advances like novel lipid probes and microscopy techniques to visualize and study lipids *in vivo* will be useful to overcome these limitations.

## Abbreviatons:

Aβ: – amyloid beta;
AD: – Alzhheimer's disease;
APP: – amyloid precursor protein;
ASM: – acid SMase;
C1P: – Cer-1-phosphate;
Cer: – ceramide;
EAP: – ethanolamine phosphate;
ER: – endoplasmic reticulum;
GABARAP: – γ-aminobutyric acid receptor-associated protein;
GBA: – glucocerebrosidase;
GCS: – GlcCer synthase;
GlcCer: – glucosylceramide;
GUV: – giant unilamellar vesicle;
HD: – Huntington's disease;
iPSC: – induced pluripotent stem cell;
KO: – knockout;
LC3: – light chain 3;
LSD: – lysosomal storage disorder;
LYNUS: – lysosomal nutrient-sensing complex;
MTM: – myotubularin phosphatase family;
mTOR: – mammalian target of rapamycin;
PD: – Parkinson's disease;
PE: – phosphatidylethanolamine;
PI: – phosphatidylinositol;
PI3K: – PI-3 kinase;
PI3P: – PI-3 phosphate;
PICALM: – PI binding clathrin assembly protein;
polyQ: – polyglutamine;
S1P: – sphingosine-1-phosphate;
SM: – sphingomyelin;
SMase: – sphingomyelinase;
Sph: – sphingosine;
SphK: – Sph kinase;
TFEB: – transcription factor EB.
